# Chronic myeloid leukemia (CML): association of treatment satisfaction, negative medication experience and treatment restrictions with health outcomes, from the patient’s perspective

**DOI:** 10.1186/1477-7525-11-167

**Published:** 2013-10-08

**Authors:** Ishan Hirji, Shaloo Gupta, Amir Goren, Diana R Chirovsky, Alyson B Moadel, Eduardo Olavarria, Timothy W Victor, Catherine C Davis

**Affiliations:** 1 Global Health Economics and Outcomes Research, Bristol Myers Squibb, Wallingford, CT, USA; 2 Health Outcomes Practice, Kantar Health, Princeton, NJ, USA; 3 Health Outcomes Practice, Kantar Health, New York, NY, USA; 4 Department of Health Policy and Management, University of North Carolina at Chapel Hill, Chapel Hill, NC, USA; 5 Albert Einstein College of Medicine, Bronx, NY, USA; 6 Hospital de Navarra, Pamplona, Spain; 7 Global Health Economics and Outcomes Research, Bristol Myers Squibb, Princeton, NJ, USA

**Keywords:** CML, Adherence, Quality of life, Treatment satisfaction, Treatment restrictions

## Abstract

**Background:**

The availability of the tyrosine-kinase inhibitor (TKI), imatinib, and later introduction of second generation TKIs, dasatinib and nilotinib, have not only improved clinical outcomes of patients with chronic myeloid leukemia (CML), but also provide multiple therapeutic options for CML patients. Despite the widespread use of these oral therapies, little is known about the impact of different treatment regimens on patient-reported outcomes (PROs) among CML patients. The objective of this study was to assess the impact of patient-reported treatment restrictions and negative medication experiences (NMEs) on satisfaction and other health outcomes among patients with CML treated with oral TKIs.

**Methods:**

Participants recruited from survey panels and patient networks in the United States (US) and Europe completed an online questionnaire. Respondents included adults (≥18 years) with chronic-phase CML currently on TKI treatment. Study variables included treatment difficulty (i.e., difficulty in following treatment regimens), CML dietary/dosing requirements, NMEs, and validated PROs assessing treatment satisfaction, health-related quality of life (HRQoL), activity impairment, and non-adherence. Structural equation models assessed associations among variables, controlling for covariates.

**Results:**

303 patients with CML (US n=152; Europe n=151; mean age 51.5 years; 46.2% male) completed the questionnaire. Approximately 30% of patients reported treatment difficulties; treatment difficulty was higher among nilotinib (63.3%) than among dasatinib (2.6%) or imatinib (19.2%) treated patients (p<0.0001). Non-adherence was generally low; however, patients on nilotinib vs. imatinib reported missing doses more often (p<0.05). Treatment satisfaction was associated with significantly increased HRQoL (p<0.05) and lower activity impairment (p<0.01). NMEs were associated with decreased treatment satisfaction (p<0.01) and HRQoL (p<0.05), and greater activity impairment (p<0.01). Higher overall treatment restrictions were associated with greater treatment difficulty (p<0.001), which correlated with non-adherence (p<0.01).

**Conclusions:**

Treatment satisfaction and NMEs are important factors associated with HRQoL among patients with CML. Increased treatment restrictions and associated difficulty may affect adherence with TKIs. Choosing a CML treatment regimen that is simple and conveniently adaptable in patients’ normal routine can be an important determinant of HRQoL and adherence.

## Background

Chronic myeloid leukemia (CML), a rare cancer, accounts for approximately 15% of leukemia cases [1]. The estimated number of new CML cases per year in the United States (US) is 5,430, or 1.6 per 100,000 population in 2012 [2]. In Europe, crude incidence of CML is estimated to be 0.92 per 100,000 [3].

CML cells contain a BCR-ABL gene, not typically found in normal cells, that produces a protein (BCR-ABL) causing CML cells to proliferate [4]. CML occurs in three phases: chronic, accelerated, and blast crisis. Disease staging is primarily based on percent of blasts in the blood and bone marrow. Most cases of CML are diagnosed in chronic phase (CP) [4]. In CML-CP patients usually have <10% blasts in their blood samples and the disease can be controlled effectively with standard treatment, with patients experiencing few or no symptoms [5,6]. Symptoms can include anemia-related fatigue, abdominal pain, bleeding, bruising, and enlarged lymph nodes. Accelerated phase patients are defined as experiencing 10-19% blasts [7] and patients progressing from CP to accelerated phase CML experience a reduction in red blood cells and platelets, fluctuations in white blood cell count, an increase in blast cells, and swelling of the spleen. According to the World Health Organization, the definition of CML-blast phase includes patients who have ≥20% blasts, while the blast crisis phase [7] differs from the acceleration phase in that 30% or more blast cells are found in the blood or bone marrow, and patients may experience swelling of the liver, in addition to symptoms present during earlier phases [4]. The blast crisis phase is typically fatal [4].

A major objective in CML clinical management is to prevent progression from chronic to accelerated and blast crisis phases. While earlier treatments, such as cytoreductive chemo- and interferon therapies increased overall survival rates among patients, the advent of oral tyrosine-kinase inhibitors (TKIs) has changed the CML treatment landscape [8]. TKIs target the BCR-ABL protein in CML cells and typically do not influence normal cells, and they are usually associated with fewer side effects compared with chemotherapy drugs or interferon therapy [9]. Clinical trials of the first-generation TKI, imatinib, demonstrate 5-year survival rates of at least 89% in patients in CP, transforming CML into a chronic disease for many patients [8,10]. Second-generation oral TKIs, dasatinib [11,12] and nilotinib [13], have provided additional treatment options with superior cytogenetic response compared with imatinib in clinical trials.

Bone marrow transplant (BMT) is considered a viable treatment alternative for only a small subset of pediatric and young adult chronic phase CML patients due to the high risk of mortality and other serious complications that can occur with this procedure [6]. Difficulties in procuring suitable donors further preclude the broader application of BMT for the treatment of CML [6]. In patients for whom CML has progressed to either the accelerated or blast crisis phases, however, allogeneic hematopoietic stem cell transplantation is the primary recommended treatment option [14].

Imatinib, dasatinib, and nilotinib are generally well tolerated, but each has also been associated with side effects that could potentially affect its use. Common side effects for all three TKIs include nausea, fatigue, and rashes [15]. Other side effects, including diarrhea, fluid retention events, and headaches, have also been reported [16-18]. Occurrence and persistence of such side effects likely affect a patient’s perceived experience with CML medication, potentially reducing adherence, which consequently, may affect treatment outcomes [15].

With the widespread use of these oral TKIs in CP-CML, burden of treatment administration has decreased compared with previously used chemotherapy regimens; however, daily administration is required for effective management of CML [19]. Therefore, treatment satisfaction, symptom, and/or side effect management become important determinants of health-related quality of life (HRQoL) and TKI treatment adherence. Furthermore, understanding the overall patient experience with the three TKIs is important given differences in labeled dosing and administration requirements with respect to meal timing and frequency of dosing [16-18].

The need to measure patient-reported outcomes (PROs) to help guide treatment decisions, especially for currently available TKIs, has been highlighted in the literature [20]. However, few studies have assessed PROs in a real-world setting [21], and the effects of long-term oral CML treatment on patient-reported HRQoL have not been explored comprehensively [6]. Efficace et al. [22] found that patients with CML on long-term imatinib therapy exhibited HRQoL comparable to that of the general population. Younger (aged 18–39) vs. older patients experienced significant activity limitations resulting from physical and emotional impairments. Likewise, female vs. male patients experienced lower HRQoL, indicating fatigue as the most frequent symptom [22].

This study was conducted to better understand the impact of different dosing aspects of TKI treatment on treatment satisfaction, HRQoL, adherence, and activity impairment. The primary objective was to assess, from the patient’s perspective, the impact of CML treatment restrictions, dosing administration, and negative medication experiences (NMEs) on overall treatment satisfaction for each of the three oral TKIs (imatinib, dasatinib, and nilotinib). The study further explored the impact of treatment satisfaction and NMEs on adherence, HRQoL, and activity impairment. A secondary objective was to examine the impact of different treatment restrictions on overall patient-reported difficulty in following an oral TKI regimen and its impact on patient-reported non-adherence among patients with CML.

## Methods

### Data source and sample

The study population consisted of 303 patients reporting a physician diagnosis of CML in the US and Europe (UK, France, Germany, Spain, and Italy). Ninety-seven respondents (32%) in the US were identified using NexCura’s recruitment services. NexCura maintains a database of more than 1,000,000 patients with cancer, cardiovascular, and respiratory diseases. NexCura provides educational tools to patients visiting health information websites (e.g., the American Cancer Society [cancer.org]). All potential panelists must register with the panel through a unique email address and password and complete an in-depth demographic registration profile they routinely update. Participants of the current study were sent an invitation to participate in the web-based questionnaire. An additional 55 US respondents (18.2%) and all 151 (49.8%) European respondents were recruited via telephone through interview facilities, including panel recruiting, grassroots campaigns, and newspaper advertising and physician referrals of patients diagnosed with CML.

Eligible study participants included adults (≥18 years) with self-reported diagnosis of CP-CML who were currently on, or on a drug holiday from, oral TKI treatment (imatinib, dasatinib, or nilotinib). Respondents were excluded if they were: 1) currently in accelerated or blast phase of CML or not sure of their current CML phase, 2) not currently being treated with an oral TKI, with no intent of initiating or resuming treatment, or 3) unsure about their current treatment. To ensure PROs appropriately represented the treatment experience of patients with CML, patients diagnosed with any cancer other than CML were excluded.

Data were collected from November 2010 through May 2011 via an online patient questionnaire. The questionnaire was developed with input from an advisory board comprising opinion leaders and health care professionals from the US (n=3) and Europe (n=2). The advisory board independently reviewed the item pools and helped establish face validity and content validity for the patient questionnaire. The patient questionnaire was also piloted in a sub-sample of patients with CP-CML (n=20). The 20 patients with CML participated in an in-depth telephone interview (approximately 45 minutes in length). To ensure consistency in the tone and presentation of the questions, a single moderator conducted all 20 interviews. Data generated from a literature review and the pilot interviews were used to identify the key constructs to be included in the final survey instrument. The study was reviewed and approved by Essex IRB (Lebanon, NJ), and all study participants provided their informed consent prior to participation. Participants were provided with incentives for their completion of the survey.

The questionnaire incorporated validated PRO instruments, plus 52 questions focused on ascertaining overall CML disease status, treatment burden, treatment satisfaction, non-adherence, and the physician-provider relationship.

### CML treatment

CML treatment-related variables included: 1) CML treatment restrictions, 2) number of CML treatment doses per day, and 3) NMEs. Treatment restrictions included specific dietary restrictions and dosing requirements and were identified according to TKI label instructions. Dietary restrictions included the need to take the CML medication while fasting (i.e., on an empty stomach), with food, without certain foods (i.e., grapefruit juice), with water, dissolved in water and taken immediately, or without certain non-CML medications. Dosing requirements included taking the medication once each day, twice each day, taking two or more pills at one time, and taking pills at specific times of day or at specific hours apart (e.g., 12 hours).

To quantify the incremental burden associated with increased CML treatment doses per day, a continuous variable was used to indicate the number of times a day an oral TKI was taken, measured using a single item: “Thinking about a typical day, how many times do you take your current CML treatment?”

To identify NMEs associated with TKI treatment, patients were asked to identify whether they experienced any of 34 symptoms associated with CML disease and treatment in the past 4 weeks. If participants reported experiencing at least one NME in the past 4 weeks (experience component), they were further asked to identify which NMEs were the most bothersome (Yes/No; bothersome component), and which NMEs had the biggest impact on patients’ ability to go about their normal routine (Yes/No; impact component).

### Satisfaction with treatment and health outcomes

Overall satisfaction with current CML treatment was assessed using the Satisfaction with Therapy component of the Cancer Therapy Satisfaction Questionnaire (CTSQ) [23,24]. Respondents were asked about the difficulty of following specific CML treatment restrictions or requirements: “Would it be easier for you to follow your CML treatment regimen if you did not need to follow the specific dietary restrictions or requirements?” (Yes/No). Individuals who did not report any treatment restrictions did not complete this questionnaire item but were coded as “No” responses, reflecting the lack of possibility of improvement over their current regimen.

HRQoL was assessed using Physical and Mental Component Summary (PCS, MCS) scores from the Medical Outcomes Study 12-Item Short Form Survey Instrument (SF-12v2), a multipurpose, generic HRQoL instrument [25]. The SF-12v2 is a validated, shortened version of the SF-36v2, which has been used previously as an HRQoL measure by Efficace et al. in patients with CML [22]. The SF-12v2 was deemed the appropriate choice for the current study, limiting the length of the overall survey while obtaining reliable generic measures of mental and physical health status and overall HRQoL on a scale that can be understood and compared across various comorbid conditions and populations. MCS and PCS scores have a normed mean of 50 and a standard deviation of 10 for the U.S. population. Health utilities were calculated using the SF-6D algorithm, which provides a preference-based single index measure for health, referenced to general population values, with interval scores on a theoretical 0–1 scale [26]. Activity impairment was assessed using the Work Productivity and Activity Impairment (WPAI) validated instrument [27], which consists of four subscales, generated in the form of percentages, with higher values indicating greater impairment. As data for absenteeism, presenteeism, and overall work impairment subscales were available only for employed respondents, only activity impairment was included in the analyses.

### CML treatment adherence

To understand how well respondents adhered to their current medication, patient-reported non-adherence was assessed: “In the past 4 weeks, how many doses of your current CML treatments have you missed, intentionally or unintentionally?” Similar questions ascertained how many doses patients skipped and took less than the product label-prescribed amount in the past 4 weeks. Higher number of doses missed, skipped, or taken in reduced amounts indicated a greater degree of non-adherence to CML treatment.

### Control variables

Additional analytical control variables included the number of non-CML medications taken per day (i.e., treatment for other diseases) and patient demographics and health characteristics. The number of non-CML medications taken per day was evaluated by one item: “Excluding your CML treatments, how many medications for all other conditions do you take on a daily basis? Include both prescription and over-the-counter medications.” Patient demographics and health characteristics included gender, age, time since CML diagnosis by a physician, time on current treatment, and self-reported co-morbidities (the complete list of co-morbidities included 32 conditions, which could be a result of treatment, disease complications, neither, or both).

### Statistical analyses

Participants were initially categorized according to self-reported CML treatment (imatinib, dasatinib, nilotinib, or other/decline to answer). Descriptive analyses of patient demographics and health characteristics were performed for the full study sample. Bivariate analyses for differences across patients’ current CML treatment were evaluated using Chi-square tests for categorical variables and independent sample t-tests for continuous variables.

#### Structural equation modeling

A multivariate model was developed, using structural equation modeling (SEM), to assess interrelationships among the main explanatory CML treatment-related variables, treatment satisfaction, and identified PRO variables. To explore the secondary objective, an additional model was developed, assessing the associations between treatment restrictions, treatment difficulty, and non-adherence.

Non-adherence was modeled as an unobserved latent factor, represented by missing, skipping, and taking fewer doses than recommended. NMEs were also modeled as a latent factor, represented by impact, bothersome, and experience subcomponents. Modeling these as latent factors allowed for an examination of the degree to which latent factors were represented by their components.

Standardized estimates for these models indicated direction and strength of association between: 1) composite variables and their individual components (e.g., the degree to which observed components reflect the latent factor of non-adherence), and 2) explanatory and outcome variables (i.e., as in most regression models). Regression estimates are interpretable in a similar manner as correlation coefficients (range of −1 to +1, with 0 indicating no relationship). Straight arrows in the model represent (causal) regression estimates, and curved arrows represent correlations (associations). P-values, based upon unstandardized estimates, indicate statistical significance of those estimates.

#### Item response theory modeling

To help simplify and consolidate complex information for CML treatment restrictions and the three subgroups of NMEs, given the large groups of interrelated items composing each variable, item response theory (IRT) models were used to create summary scores. IRT is a statistical theory that describes the association between where a respondent lies on the continuum of some unobserved variable and the probability of a particular item response [28,29]. Three separate IRT models were created for NMEs, reflecting the experience, bothersome, and impact items to define a higher-order NMEs variable used in SEM. An IRT model was created for CML treatment restrictions defined above using the items for treatment restrictions and dosing requirements, where higher scores indicated greater restrictiveness.

#### Model fit assessments

The structural equation models were developed and tested using Mplus version 6.1 [30], and IRT models were created using R software (http://www.R-project.org). Acceptability of the overall fit of the SEM regressions was assessed using the Chi-square test and values of the Comparative Fit Index (CFI), Tucker-Lewis Index (TLI), and Root Mean Square Error of Approximation (RMSEA) statistics [31-33]. For the Chi-square test, good model fit was demonstrated by Chi-square values divided by degrees of freedom (relative Chi-square) that were close to or lower than 2, and non-significant associated p-values. Higher CFI and TLI values approaching 1 indicated a better fit (e.g., 0.95 and higher), whereas lower RMSEA values (i.e., below 0.06) were considered a good model fit.

## Results

### Baseline demographics and clinical characteristics

The study population consisted of 303 adults diagnosed with CML across the US (n=152), United Kingdom (n=25), France (n=24), Germany (n=33), Italy (n=37), and Spain (n=32). Among all respondents, 68.6% reported current treatment as imatinib (US: n=102, EU: n=106), 12.5% dasatinib (US: n=25, EU: n=13), 16.2% nilotinib (US: n=22, EU: n=27), and 2.7% reported other/decline to answer (US: n=1, EU: n=3) (Table 1). Mean age was 51.5 years (SD=13.6), 46.2% were male, and mean time since diagnosis was 4.8 years (SD=4.5), with 12.3% reporting being within 12 months of diagnosis. One respondent was missing information on length of time since diagnosis and was excluded from analyses involving this variable. Mean number of comorbidities was 1.4 (SD=2.4), with the most common being hypertension (16.2%), insomnia/sleeping difficulties (10.6%), and depression (8.3%). In the US the average age was 51.5 years (SD=11.9); 38.2% were male and the average time since diagnosis was 4.8 years (SD=3.8). In Europe the average age was 51.6 years (SD=15.1); 54.3% were male, and the average time since diagnosis was 4.9 years (SD=5.1). All other US and EU patient characteristics and clinical measures were similar for respondents (data not shown).

**Table 1:** Demographics and clinical characteristics of CML cohort

	**Total CML-CP Patients (n=303)**
Gender (n, %)	
Male	140 (46.2%)
Female	163 (53.8%)
Age (mean years, SD)	51.5 (±13.6) years
Time since CML diagnosis (mean years, SD)	4.8 (±4.5) years
Time since CML diagnosis (n, %)	
<2 years	76 (25.1%)
2 to <5 years	90 (29.7%)
≥ 5 years	136 (44.9%)
Unknown	1 (0.3%)
Country (n, %)	
USA	152 (50.2%)
Italy	37 (12.2%)
Germany	33 (10.9%)
Spain	32 (10.6%)
United Kingdom	25 (8.3%)
France	24 (7.9%)
Time on Current CML Treatment (median years, IQR)	2.75 (4.8)
Current CML Treatment (n, %)	
Imatinib	208 (68.6%)
Nilotinib	49 (16.2%)
Dasatinib	38 (12.5%)
Other	4 (1.3%)
Decline to answer	4 (1.3%)
No. of Comorbidities (mean, SD)	1.4 (±2.4)
Comorbidities (n, %)	
Hypertension	49 (16.2%)
Insomnia/sleep difficulties	32 (10.6%)
Depression	25 (8.3%)
Arthritis	24 (7.9%)
Anxiety	24 (7.9%)
Anemia	21 (6.9%)

Note: CML=Chronic Myeloid Leukemia, CP=Chronic Phase, SD=Standard Deviation, IQR=Inter Quartile Range.

### CML treatment restrictions

Fewer patients on dasatinib reported having to take their pills with food (36.8% vs. imatinib: 70.2%; p<0.01) or water (39.5% vs. imatinib: 63.0% & nilotinib: 65.3%; p<0.02; Table 2). Patients on nilotinib reported the following treatment restrictions more frequently than patients on dasatinib and imatinib: take medication while fasting (p<0.01), at specific hours apart (p<0.01), not take with certain foods (p<0.01), take twice each day (p<0.01), and take two or more pills at one time (p<0.01). Patients on nilotinib also reported taking their pills with food less frequently than patients on dasatinib (4.1% vs. 36.8%; p<0.01).

**Table 2:** CML treatment restrictions, negative medication experiences, and overall disease burden, according to current CML treatment

	**Imatinib**		**Dasatinib**		**Nilotinib**		**Imatinib vs. Dasatinib**	**Imatinib vs. Nilotinib**	**Dasatinib vs. Nilotinib**
	**(N=208)**		**(N=38)**		**(N=49)**				
	**N**	**%**	**N**	**%**	**N**	**%**	**p-value**	**p-value**	**p-value**
Treatment Restrictions									
*Dietary Restrictions*									
While fasting	5	2.40%	0	0.00%	42	85.71%	0.33	<.01	<.01
With food	146	70.19%	14	36.84%	2	4.08%	<.01	<.01	<.01
Without certain foods	88	42.31%	16	42.11%	36	73.47%	0.98	<.01	<.01
With water	131	62.98%	15	39.47%	32	65.31%	<.01	0.76	0.02
Dissolve tablets in water and drink immediately	3	1.44%	0	0.00%	1	2.04%	0.46	0.76	0.38
Without other non-CML medications	22	10.58%	2	5.26%	9	18.37%	0.31	0.13	0.07
*Dosing Requirements*									
Once each day	105	50.48%	17	44.74%	3	6.12%	0.52	<.01	<.01
Twice each day	15	7.21%	5	13.16%	32	65.31%	0.22	<.01	<.01
Two or more pills at one time	26	12.50%	3	7.89%	24	48.98%	0.42	<.01	<.01
At specific times of the day	101	48.56%	19	50.00%	31	63.27%	0.87	0.06	0.22
At specific hours apart	32	15.38%	7	18.42%	38	77.55%	0.64	<.01	<.01
	**Mean**	**SD**	**Mean**	**SD**	**Mean**	**SD**	**p-value**	**p-value**	**p-value**
Treatment Restriction Score	−0.06	0.69	−0.31	0.77	0.61	0.68	0.06	<.01	<.01
No. Treatment Doses (times per day)	1.20	0.50	1.32	0.62	1.88	0.39	0.27	<.01	<.01
Negative Medication Experience									
Experienced Score	0.06	0.90	−0.10	0.98	0.07	0.91	0.35	0.96	0.42
Bothersome Score	0.02	0.70	0.10	0.62	0.07	0.74	0.47	0.66	0.83
Impact Score	0.02	0.66	0.10	0.57	0.11	0.65	0.45	0.38	0.93
No. Non-CML Medications per day	2.02	2.67	2.47	4.07	2.27	2.77	0.52	0.58	0.79
Time since CML diagnosis (years)	4.85	4.31	5.32	4.47	3.69	4.14	0.55	0.09	0.09
Time on Current CML Treatment (years)	4.60	2.98	2.07	1.69	1.55	1.35	<.01	<.01	0.12
No. Comorbidities	1.17	2.13	1.97	3.19	1.59	2.71	0.14	0.32	0.56

Note: CML=Chronic Myeloid Leukemia, No.=Number of, SD=Standard Deviation.

Based on the IRT model, the requirements of taking medication with water or food were at the low end of the restrictions scale (representing “easier” items), whereas the requirements of not taking medication with other non-CML medication or taking medication while fasting were at the high end of the scale (representing more “difficult” items). Overall CML treatment restriction scores ranged from −1.33 to 1.60, with positive values indicating greater restrictiveness. Average scores were low among patients on dasatinib (Mean=−0.31, SD=0.77) and patients on imatinib (Mean=−0.06, SD=0.69), whereas average scores were significantly higher among patients on nilotinib (Mean=0.61, SD=0.68; p<0.01).

### Number of CML treatment doses (times per day)

The dosing regimens approved by the Food and Drug Administration (FDA) are 2 per day for nilotinib, 1 per day for dasatinib, and either 1 or 2 per day for imatinib [16-18]. Consistent with these guidelines, patients on nilotinib reported having to take their CML medication significantly more times per day (Mean=1.88, SD=0.39), compared with patients on dasatinib (Mean=1.32, SD=0.62) and imatinib (Mean=1.20, SD=0.50; p<0.01).

### NMEs

No significant differences were found across the three TKIs on the average scores for experiencing NMEs or perceiving them as being bothersome or having an impact on patients’ daily routine (Table 2).

### Control variables

There were no significant differences in the total number of non-CML medications taken per day, time since CML diagnosis, and the number of comorbidities across the three TKI groups. Patients on imatinib had used their current treatment for a significantly longer duration (Mean=4.60 years, SD=2.89) than patients on dasatinib (Mean=2.07 years, SD=1.69) or nilotinib (Mean=1.55 years, SD=1.35; p<0.01).

### Treatment difficulty

Significantly more patients on nilotinib reported having difficulty taking their CML medication (n=31, 63.3%), compared with patients on imatinib (n=40, 19.2%; p<0.01) and dasatinib (n=1, 2.6%; p<0.01) (Table 3). Patients on dasatinib represented the smallest proportion of those reporting having difficulty taking their CML medication (p≤0.01 vs. imatinib and nilotinib).

**Table 3:** Treatment difficulty and non-adherence among CML patients, according to current CML treatment

	**Imatinib**		**Dasatinib**		**Nilotinib**		**Imatinib vs. Dasatinib**	**Imatinib vs. Nilotinib**	**Dasatinib vs. Nilotinib**
	**(N=208)**		**(N=38)**		**(N=49)**				
	**Mean, N**	**SD, %**	**Mean, N**	**SD, %**	**Mean, N**	**SD, %**	**p-value**	**p-value**	**p-value**
Treatment Difficulty									
Yes	40	19.23%	1	2.63%	31	63.27%	0.01	<.01	<.01
No	168	80.77%	37	97.37%	18	36.73%	0.01	<.01	<.01
Non-Adherence									
No. Missed Doses	0.45	1.08	0.63	1.73	1.02	1.60	0.54	0.02	0.29
No. Skipped Doses	0.41	1.53	0.53	1.83	0.73	1.62	0.72	0.21	0.58
No. Doses Less than Prescribed	0.10	0.52	1.05	4.78	0.47	1.46	0.23	0.08	0.47

Note: CML=Chronic Myeloid Leukemia, No.=Number of, SD=Standard Deviation.

### Satisfaction with CML treatment, HRQoL, and activity impairment

CTSQ results indicated that CML patients were generally satisfied with their treatment (total, Mean=83.7, SD=14.4). No significant differences emerged for satisfaction with treatment across the three TKIs. Similarly, no significant differences emerged for MCS, PCS, health utilities, and activity impairment, across TKIs (data not shown).

### CML treatment adherence

The majority of patients reported being adherent to their CML treatment, with 34% (n=103) reporting having missed (unintentional) or skipped (intentional) doses, or having taken fewer doses than prescribed within the past 4 weeks. However, patients on nilotinib (Mean=1.02, SD=1.60) reported missing their doses significantly more often than imatinib users (Mean=0.45, SD=1.08; p<0.05).

### Multivariate models using SEM for total sample (n=302)

**Figure 1:**
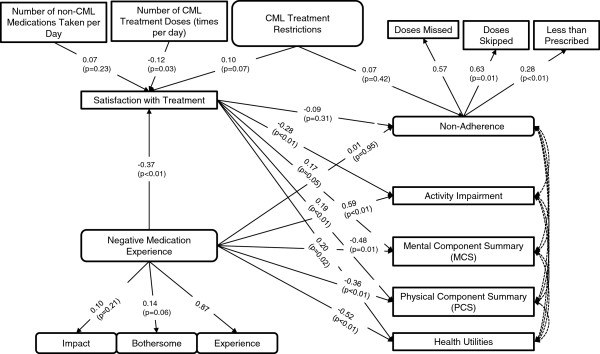
**Multivariate structural equation model predicting the associations of CML treatment restrictions, treatment doses, NME, CML treatment satisfaction, and patient-reported outcomes among CML patients.** Note. The following covariates or non-significant predictors (of adherence, activity impairment, MCS, PCS, and health utilities) are not presented: age, gender, number of comorbidities, time since diagnosis, and number of non-CML medications taken per day. Straight lines indicate betas and curved lines indicate correlations. Residual error terms, estimated for all predicted variables and factors, are not presented; neither are correlation estimates. Non-parenthetical values are standardized estimates (ranging from −1 to +1, with 0 = no effect) indicating strength and direction of association; these values can be compared across predictors. P-values are based on the unstandardized path estimates. No p-values are available for the first indicators per factor, which set the scale. Fit statistics indicate a fair fit of the data to the model (χ^2^ test of model fit=408.8; df=76; p-value<0.01. CFI=0.79; TLI=0.63; RMSEA=0.12). CML = chronic myeloid leukemia, NME = negative medication experience.

SEM results examining the associations of CML treatment restrictions, dosing requirements, NMEs, CML treatment satisfaction, and PROs are shown in Figure 1. Fit statistics indicate a fair fit of the model to the data (χ^2^=408.8, degrees of freedom=76, p-value<0.01; CFI=0.79; TLI=0.63; RMSEA=0.12).

CML treatment restrictions were not significantly associated with self-reported non-adherence (beta=0.07, p=0.42) or satisfaction with treatment (beta=0.10, p=0.07). However, the number of CML treatment doses (times per day) were significantly negatively associated with treatment satisfaction (beta=−0.12, p=0.03), indicating that as the number of treatment doses increased, satisfaction with treatment decreased.

Satisfaction with treatment was a direct predictor of higher PCS (beta=0.19, p<0.01), MCS (marginally, at p=0.05), and health utility scores (beta=0.20, p=0.02), and lower activity impairment (beta=−0.28, p<0.01), adjusting for the effect of age, gender, co-morbidities, time since diagnosis, and non-CML medications. Satisfaction with treatment was not a significant predictor of non-adherence (p=0.31).

NME was a strong predictor of lower satisfaction with treatment (beta=−0.37, p<0.01), PCS (beta=−0.36, p<0.01), MCS (beta=−0.48, p=0.01), and health utility scores (beta=−0.52, p<0.01), and greater activity impairment (beta=0.59, p<0.01), after adjusting for aforementioned covariates. NME was not a significant predictor of non-adherence (p=0.95). There were strong direct, deleterious effects of NME on the PROs, as well as more moderate indirect (mediated) effects via satisfaction with treatment that accounted for at least 10% of the total relationship between NME and the PROs.

The model covariates—age, gender, number of comorbidities, years since diagnosis, and number of non-CML medications—were not significantly associated with non-adherence (p≥0.23 for all; data not shown).

**Figure 2:**
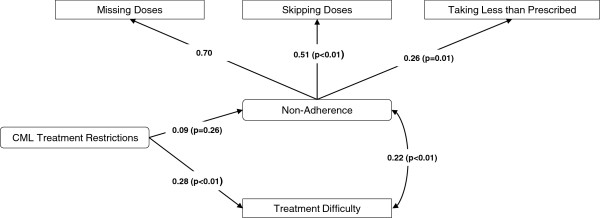
**Multivariate structural equation model predicting the associations of CML treatment restrictions, patient reported difficulty following CML treatment, and non-adherence among CML patients.** Note. Straight lines indicate betas and curved lines indicate correlations. Residual error terms, estimated for all predicted variables and factors, are not presented. Non-parenthetical values are standardized estimates (ranging from −1 to +1, with 0 = no effect) indicating strength and direction of association; these values can be compared across predictors. P-values are based on the unstandardized path estimates. No p-values are available for the first indicators per factor, which set the scale. Fit statistics indicate a good fit of the data to the model (χ^2^ test of model fit=91.6; df=10; p-value<0.01. CFI=1.0; TLI=1.01; RMSEA<0.01). CML = chronic myeloid leukemia.

A second multivariate model, also using SEM, further examined inter-relationships between CML treatment restrictions, patient-reported treatment difficulty, and non-adherence (Figure 2). Given that none of the abovementioned covariates were significantly associated with non-adherence in the first model, these were excluded to retain maximum statistical power. Fit statistics indicated a good fit of the model to the data, given the high CFI and TLI values and a small RMSEA value (χ^2^=91.6, degrees of freedom=10, p-value<0.01; CFI=1.00; TLI=1.01; RMSEA<0.01).

Higher overall CML treatment restriction scores were associated with significantly greater difficulty in taking medication as prescribed (beta=0.28, p<0.01). Self-reported treatment difficultly and non-adherence were highly positively correlated (r=0.22, p<0.01). Therefore, as treatment difficulty increased, non-adherence increased. CML treatment restrictions were not significantly, directly associated with non-adherence (p=0.26).

## Discussion

Oral TKIs have revolutionized treatment options for patients with CML, enabling a shift in focus from improving survival to improving PROs. The present study is one of the few that evaluate how CML treatment characteristics and symptoms can affect outcomes from the patient’s perspective. After adjusting for patient characteristics, patients’ negative experience with their CML medication was a strong determinant of their overall treatment satisfaction. Additionally, greater NME and lower satisfaction with treatment were associated with significantly increased activity impairment. Furthermore, lower NME and greater treatment satisfaction were associated with significantly greater HRQoL. Considering that patients with CML are required to take daily, life-long treatment to manage their disease, optimizing satisfaction with current oral treatment options plays an important and direct role in improving the well-being of patients with CML.

Whereas CML treatment restrictions were not significantly associated with treatment satisfaction and non-adherence overall, having to take CML treatments more than once a day (i.e., nilotinb and imatinib) did have a significant negative association with patient satisfaction with treatment (as noted previously, nilotinib should be taken twice per day and imatinib taken once or twice per day). Consistent with this observation, increased restrictions in CML treatment were associated with patient-reported difficulty following these treatment restrictions or requirements, which, in turn, was associated with increased reports of non-adherence (r=0.22, p<0.01) to treatment. Eliasson et al., found both intentional and unintentional reasons for non-adherence to imatinib, with patients being unaware that non-adherence could result in a negative clinical response [34]. Factors predicting adherence to imatinib therapy are group assistance, being informed, concomitant drug burden, managing side effects, and reminders to take the medicine [34-36].

Continuous and adequate dosing is essential to patient success on CP-CML therapy; however, many struggle to remain adherent to treatment. Among patients with CML, adherence to treatment correlates with the probability of achieving an improvement in long-term clinical outcomes, including major molecular response and improved event-free survival [37-39]. Therefore, choosing a regimen that can be adapted conveniently in patients’ normal routine, with minimal disturbance, is an important consideration for treatment choice and adherence.

Corresponding with general labeling instructions, patients on dasatinib were less likely to report certain dietary and dosing restrictions than patients taking nilotinib or imatinib. For example, imatinib should be taken along with food and water, whereas nilotinib should be taken at least two hours after and one hour before food consumption. Dasatinib, on the other hand, should be taken with water, but can be taken with or without food. Whereas imatinib and dasatinib can be taken once daily, nilotinib needs to be taken twice daily, approximately 12 hours apart [16-18]. As the present study mainly focused on understanding the overall treatment burden among patients with CP-CML, the study did not achieve sufficient sample size of dasatinib and nilotinib users to facilitate direct drug-to-drug comparisons of HRQoL outcomes. Nevertheless, the results of this study suggest that certain treatment restrictions and disease-related symptoms are important determinants of PROs among patients with CML. Specifically, based on the IRT model, the requirement of having to take medications while fasting (i.e., nilotinib) was considered by patients as being more “difficult,” whereas taking medications regardless of meals/water intake (i.e., dasatinb, imatinib, and nilotinib) was considered “easier.” Treatment restrictions (and corresponding treatment difficulty) are burdensome to patients, which ultimately may affect adherence. Further research is needed to understand whether differences in characteristics of available TKIs may give rise to different HRQoL outcomes across these therapies.

The present research is the first to provide a multivariate model of the complex inter-relationships between patient treatment satisfaction, disease-related symptoms, and HRQoL outcomes among a geographically diverse sample of patients with chronic-phase CML in the US and Europe. Nevertheless, the study has certain limitations. The findings may be limited by potential inaccuracies in participants’ recollections regarding medical diagnoses and other study variables. Further studies should gather more objective measures of diagnosis and treatment information from patient medical records and adherence data, to corroborate any self-reported data. The survey for the current study was administered between late 2010 and early 2011, during which time bosutinib and ponatinib did not have FDA approval for treating patients with CML; therefore, no data were available or analyzed for these new TKIs. The use of a cross-sectional design precludes the ability to draw causal inferences (for example, although it is assumed that treatment satisfaction has a more substantial effect on HRQoL than vice versa, both reverse causation and other causal explanations cannot be ruled out). It would, therefore, be instructive to perform repeated measures or longitudinal analyses not only to replicate the current findings but also to determine whether there are fluctuations in CP-CML outcomes over longer periods of time. Non-adherence was measured using three distinct questions (skipping, missing, and taking less medication than prescribed); given that these items have not been validated, further research is needed to estimate their reliability and validity vis-à-vis existing, validated measures of adherence and non-adherence. The HRQoL measure (SF-12v2) included in the current study was generic, not specific to cancer. Therefore, whereas the SF-12v2 captured the potential overall impact of treatment, it may have been relatively less sensitive to detecting cancer-specific differences and may have underestimated those effects. Lastly, there is always the possibility for omitted variable bias if important explanatory variables are not accounted for in the model. Fit statistics for the present multivariate models indicated a fair to good model fit (Figures 1, 2), suggesting that additional variability in the data may not be accounted for by the hypothesized model. Additional research is needed to better understand other characteristics that may affect PROs and non-adherence beyond those assessed in the current study.

## Conclusion

In conclusion, the results of this study demonstrate that both treatment satisfaction and NMEs with oral TKIs are important factors that can affect HRQoL among patients with CML. In addition, treatment restrictions are associated with patient-reported difficulty in following prescribed treatment regimens, which in turn, may affect adherence with TKI treatment. Continuous and adequate dosing is essential to patient success on CP-CML therapy; however, many struggle to remain adherent to treatment. Choosing an oral treatment regimen that can be adapted conveniently in patients’ normal routine may be an important determinant of HRQoL and adherence among patients with CML.

## Abbreviations

BMT: Bone marrow transplant; CFI: Comparative fit index; CML: Chronic myeloid leukemia; CP: Chronic phase; CTSQ: Cancer therapy satisfaction questionnaire; DF: degrees of freedom; EU: Europe; FDA: Food and Drug Administration; HRQoL: Health-related quality of life; IRT: Item response theory; MCS: Mental Component Summary of the SF-12v2 health survey; NME: Negative medication experience; PCS: Physical Component Summary of the SF-12v2 health survey; PRO: Patient-reported outcome; RMSEA: Root mean square error of approximation; SD: Standard deviation; SEM: Structural equation modeling; TKI: Tyrosine-kinase inhibitor; TLI: Tucker-lewis index; WPAI: Work Productivity and Activity Impairment questionnaire; US: United States.

## Competing interests

Ms. Gupta, Dr. Goren, and Dr. Victor are employees of Kantar Health who were paid consultants to BMS in connection with the development of this manuscript and the execution of the study. Ms. Hirji and Dr. Davis are employees of BMS. Ms. Chirovsky is a doctoral student at the University of North Carolina at Chapel Hill, and has received a pre-doctoral fellowship grant from BMS. Dr. Moadel and Dr. Olavarria hold consultancy roles with BMS.

## Authors’ contributions

The authors are responsible for the reported research, and have participated in the concept and design, analysis and interpretation of data, drafting or revising of the manuscript, and have approved the manuscript as submitted. Although the data are proprietary, the models and the methodology are not.
